# Large-scale uterine myoma MRI dataset covering all FIGO types with pixel-level annotations

**DOI:** 10.1038/s41597-024-03170-x

**Published:** 2024-04-22

**Authors:** Haixia Pan, Minghuang Chen, Wenpei Bai, Bin Li, Xiaoran Zhao, Meng Zhang, Dongdong Zhang, Yanan Li, Hongqiang Wang, Haotian Geng, Weiya Kong, Cong Yin, Linfeng Han, Jiahua Lan, Tian Zhao

**Affiliations:** 1 College of Software, Beihang University, Beijing, 100191 China; 2 Department of Obstetrics and Gynecology, Beijing Shijitan Hospital, Capital Medical University, Beijing, 100038 China; 3 Department of MRI/Emergency and Critical Care Medical Center, Beijing Shijitan Hospital, Capital Medical University/Peking University, Ninth Clinical Medical College, Beijing, 100038 China

**Keywords:** Reproductive disorders, Diseases

## Abstract

Uterine myomas are the most common pelvic tumors in women, which can lead to abnormal uterine bleeding, abdominal pain, pelvic compression symptoms, infertility, or adverse pregnancy. In this article, we provide a dataset named uterine myoma MRI dataset (UMD), which can be used for clinical research on uterine myoma imaging. The UMD is the largest publicly available uterine MRI dataset to date including 300 cases of uterine myoma T2-weighted imaging (T2WI) sagittal patient images and their corresponding annotation files. The UMD covers 9 types of uterine myomas classified by the International Federation of Obstetrics and Gynecology (FIGO), which were annotated and reviewed by 11 experienced doctors to ensure the authority of the annotated data. The UMD is helpful for uterine myomas classification and uterine 3D reconstruction tasks, which has important implications for clinical research on uterine myomas.

## Background & Summary

Uterine myomas, also known as myomas, uterine fibroids, or uterine leiomyoma are the most common benign tumors in women^1^. They have an incidence rate of 40%–60% in women under 35 years old, and 70%–80% in women over 50 years old^2^. Uterine myomas are generally benign and have no obvious symptoms. However, once they develop and deteriorate, they will cause menstrual bleeding (HMB), bladder dysfunction, pelvic compression, infertility, recurrent miscarriage, and other adverse consequences, and even directly threaten the patients’ life^3–5^. About 30% of reproductive-age women undergo hysterectomy due to uterine myomas^6^, which brings a great negative psychological impact on patients. Clinically, the corresponding diagnosis and treatment methods are generally determined according to the International Federation of Gynecology and Obstetrics (FIGO)^7^ classification of myomas, combined with the patient’s age, symptoms, and fertility requirements.

Magnetic resonance imaging (MRI) has the characteristics of no radiation, high soft tissue resolution, large field of view, and multi-directional imaging. It can clearly display various anatomical layers of the uterus and locate small myomas with a diameter of ≤0.3 cm^8^. MRI performs better than ultrasound (US) and computed tomography (CT) in displaying the size, location, and shape, and determining the relationship between myomas and the uterine cavity^9^. The T2WI sequence is the main inspection sequence for pelvic diseases, which can clearly display the anatomical structure of the uterus and find space-occupying lesions in the cervix and uterine cavity. The sagittal position is ideal for displaying the uterine panorama, which can well display the outline of the uterus and provide an intuitive anatomical basis for the protruding direction of uterine myomas.

Reproductive science has increasingly benefited from open science practices, including open data sharing and joint publication of derived data and their associated preprocessing pipelines. Compared with the traditional manual segmentation of organ tissues or lesion regions, learning-based segmentation algorithms can achieve faster and more objective medical image analysis to guide or support clinical decision-making. Previous research works^10–12^ have highlighted the potential of MRI image analysis and also demonstrated the feasibility of detecting and segmenting uterine myomas using a deep learning framework. Despite these encouraging results, MRI datasets for uterine myomas are still not established in routine clinical settings. Compared with the more widely used CT and structural brain imaging modalities, only a few datasets from pelvic and abdominal MRI studies are publicly accessible to relevant clinical and machine learning researchers. Image-level annotations are likely to be a major barrier to innovation and clinical translation in this field.

**Fig. 1:**
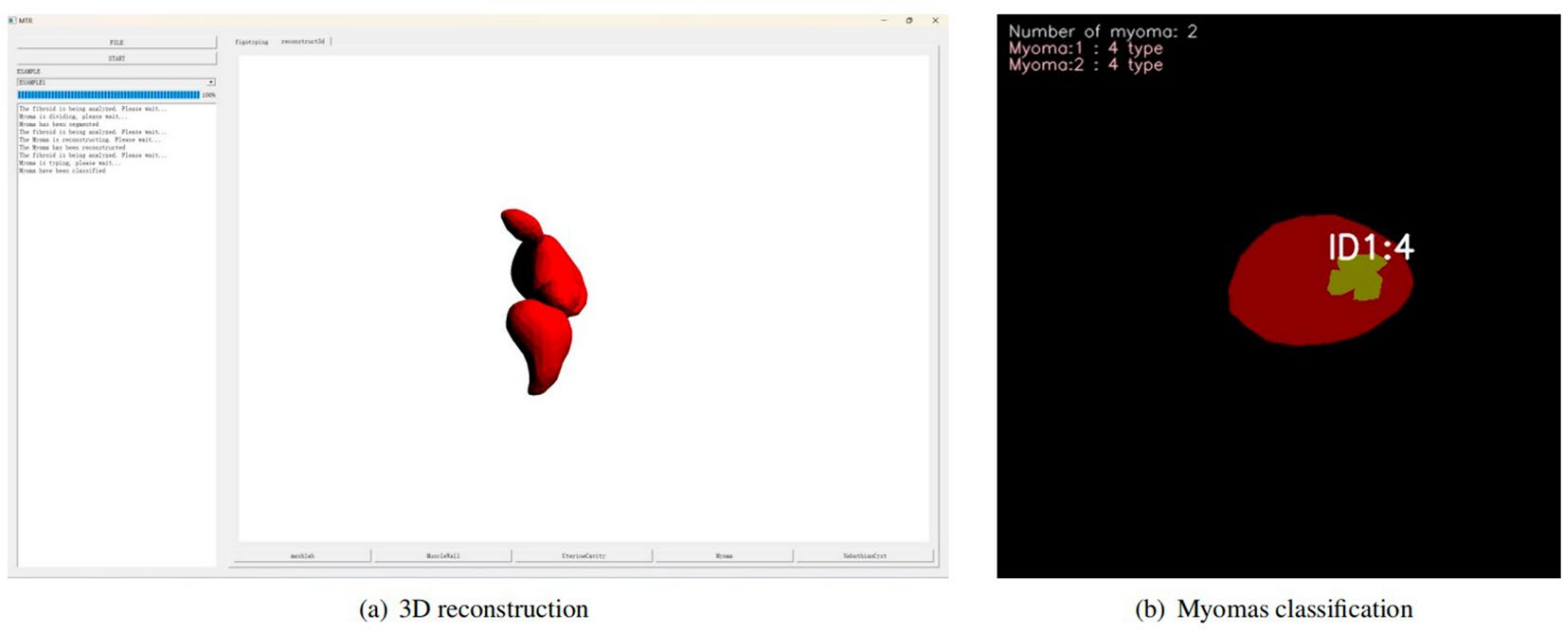
Myomas Classification and 3D Uterine Reconstruction Using UMD Dataset.

To address this limitation, we construct the Uterine Myoma MRI Dataset(UMD). The UMD provides pelvic T2WI sagittal scan images of 300 patients with uterine myoma and ROI pixel-level annotations. The uterine cavity, uterine wall, myoma, and nabothian cyst were labeled by multiple professional radiologists, consensus rule-based visual assessment of MRI performed by a wealth of clinical uterine myoma treating physicians and three radiologists, so that the quality and reliability of this dataset are further enhanced. Researchers with diverse backgrounds can use UMD for MRI imaging analysis of uterine myomas and related research based on FIGO classification standards to develop diagnosis and treatment plans. UMD can also provide data support for independent validation of artificial intelligence models developed by other research centers, promoting the drawing of increasingly reliable conclusions. Meanwhile, in our previous work^13^, we used part of the UMD case data provided in this paper to successfully construct a deep learning-based MRI intelligent diagnosis case instance segmentation model for uterine myomas. Extending this work, we also applied the UMD for myomas classification and uterine 3D reconstruction, as shown in Fig. 1. These applications enhance the clinical utility of the dataset, allowing for precise FIGO-based myoma classification and detailed 3D uterine modeling, crucial for improved diagnosis and surgical planning.

This proved the dataset we provide can be combined with artificial intelligence to further help medical institutions better analyze and manage medical resources, and improve medical level and efficiency.

In the following sections, we briefly describe the UMD acquisition and processing process, technical validation, and sharing and access policies.

## Methods

### Participants

The UMD includes 300 patients with uterine myoma who underwent pelvic magnetic resonance imaging from January 2015 to January 2023, ranging in age from 21 to 86 years old (Mean ± Standard Deviation [SD], 49.73 ± 12.96 years). This project was approved by the scientifific research ethics committee of Beijing Shijitan Hospital, Capital Medical University (code: sjtkyll-lx-2022(1)). In the approval document of the Ethics Committee, it is clearly stated that the application for exemption of informed consent for the research of this project. In addition, the release of this dataset has desensitized sensitive information and will not violate the rights and interests of patients. Meanwhile, the scientifific research ethics committee allowed for the open publication of the data.

### Image acquisition

The data collected this time were in the PHILIPS INGENIA 3.0 T ultra-high field magnetic resonance imaging system equipped with 16-channel coils. The uterine body is positioned at the center, and the scanning range covers the vagina, uterus and bilateral adnexal areas (the upper edge of the scanning range should include the bifurcation level of the iliac vessels as much as possible). Participants received T2WI scans based on sagittal, coronal, and axial scans, followed by mDIXON, DWI, and other scans. The total scan time for one case was about 25 minutes.

### Anatomical image

Uterine myomas are usually hypointense on MRI T2WI. However, in some cases, uterine myomas may show high signal intensity or a mixed signal due to degeneration and other factors. The signal’s strength is related to the water content, and the T2 signal of many lesions is stronger than that of the surrounding normal tissues, which is often highlighted. As a result, the location and size of the lesions can be clearly seen from the T2WI sequence. Acquisition of T2WI sagittal plane sequence adds windmill technology and acceleration technology such as multivalent to reduce motion artifacts and ensure image quality. To reduce the imaging time, the acceleration factor SENSE is set in the A/P direction, with a p-value of 1.3. The sagittal T2WI sequence scan parameters of the dataset in this paper are as follows, TR of 4200 ms, TE of 130 ms, an inversion angle of 90°, a voxel size of 0.8 × 0.8 × 4.0 cm3, a gap of 0.4, a field of view of 24 cm × 24 cm.

### Artefact labelling

The dataset annotation team consists of 8 clinicians with intermediate titles and more than 6 years of experience as annotation doctors, as well as 3 radiologists with over 10 years of experience as review physicians. Before conducting annotation work, the annotation doctor has received professional training, including understanding annotation specifications and familiarity with annotation tools. Initial Construction of the UMD, establish criteria for filtering the data, including inclusion and exclusion criteria. This dataset includes the following patients: (1) Female patients who have MRI findings suggestive of uterine myoma. (2)Postoperative pathology confirmed the lesion to be uterine myoma. Exclusion criteria for this dataset include (1) Female patients with female malignancies (such as cervical cancer, endometrial cancer, uterine sarcoma, ovarian cancer, etc.). (2) Other pelvic malignancies (such as rectal cancer invading the female reproductive system, patients with uterine adenomyosis, and patients during pregnancy and menstruation). (3) Data with severe respiratory artifacts and motion artifacts. (4) patients with postoperative pathology indicating non-uterine fibroid conditions. According to the aforementioned data selection criteria, 56 cases were excluded, as shown in Table 1. Finally, a total of 300 cases of uterine myoma MRI patients were included.

**Table 1:** Specific circumstances of excluded cases.

Exclusion type	Number of cases
Multiple intractable myomas	26
Myomas are too small and difficult to distinguish	7
Serious artifact	15
Low resolution	8

In order to meet clinical needs, FIGO developed a classification system for uterine myoma. This system fully considers the relationship between uterine myoma and endometrium and serosa layer, as well as the location, size, and number of uterine myomas^14^. According to the FIGO classification system, the categories are muscular wall, uterine cavity, uterine myoma, and nabothian cyst. In the labeling process, we mark the image into several regions according to the similarity or difference between the regions. The UMD provides annotations for 4 regions, namely uterine muscular wall, uterine cavity, uterine myomas, capsule,. The annotating doctors use the ITK-SNAP^15^ to outline the four regions of the muscle wall, uterine cavity (including the junctional zone), uterine myoma, and nacelle. When labeling, because there is a relationship between the muscle wall and the other three regions, the overall outline of the muscle wall should be marked first. And then mark the other parts accordingly. The data of cases whose edges of lesions could not be determined were eliminated.

**Fig. 2:**
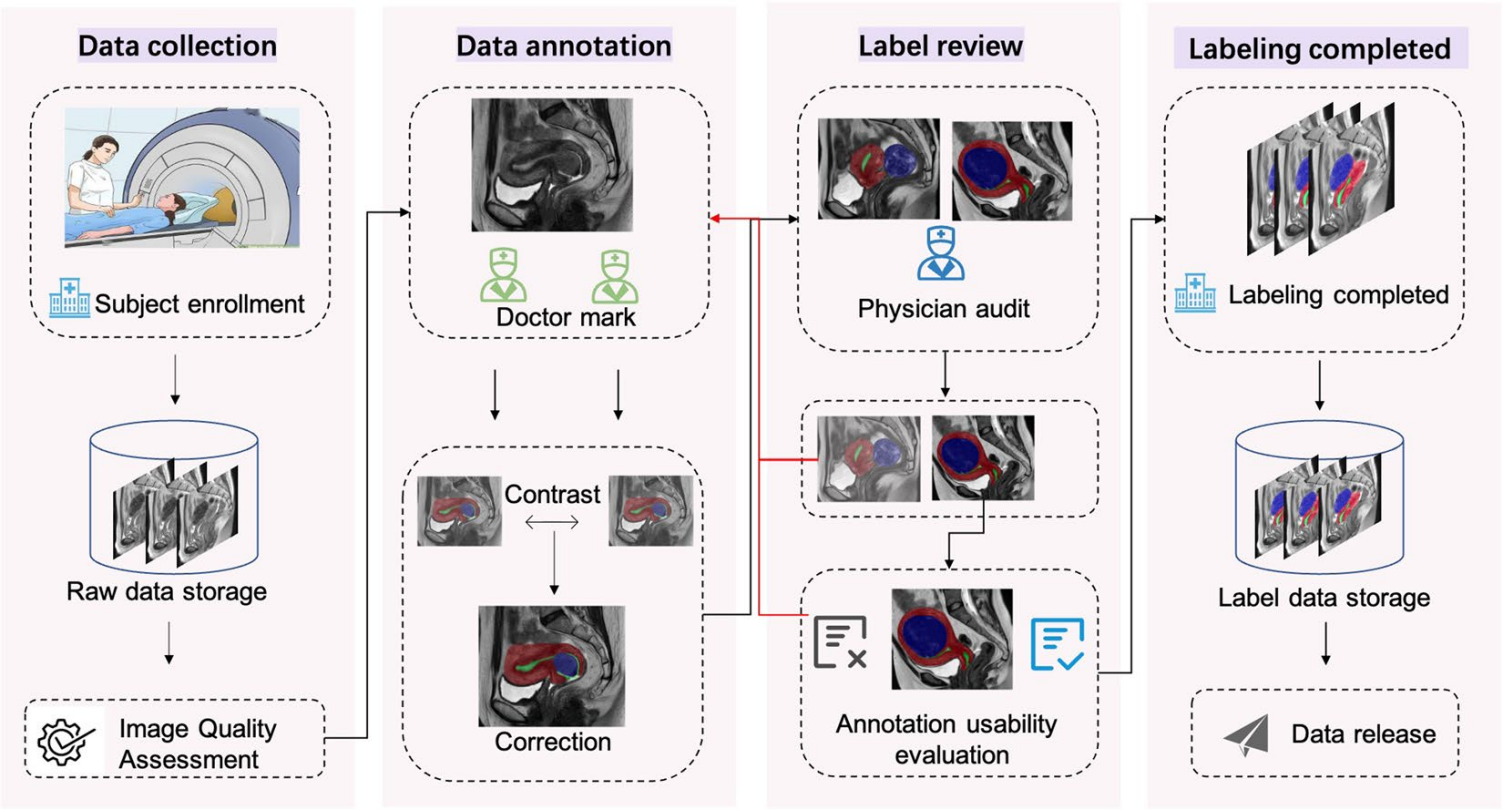
UMD dataset labeling process. Divided into data collection, data annotation, annotation review, and data archiving processes.

The UMD dataset provides annotated files for T2WI sagittal images, where annotated doctors outline the ROI area of the case. During the labeling process, if it is impossible to confirm whether it is a myoma, it will be confirmed based on slices from other sequences to ensure its reliability. At the same time, the same case will be labeled by two doctors. When these two doctors reach a consensus, the reviewing doctor will evaluate it. After the evaluation is passed, the case will be labeled. The UMD dataset construction process is shown in Fig. 2.

### Data organization

We use the ITK-SNAP tool to convert the original DICOM file to NIFTI format and use the written script file to anonymize information such as names in the DICOM file.

### Data analysis

**Fig. 3:**
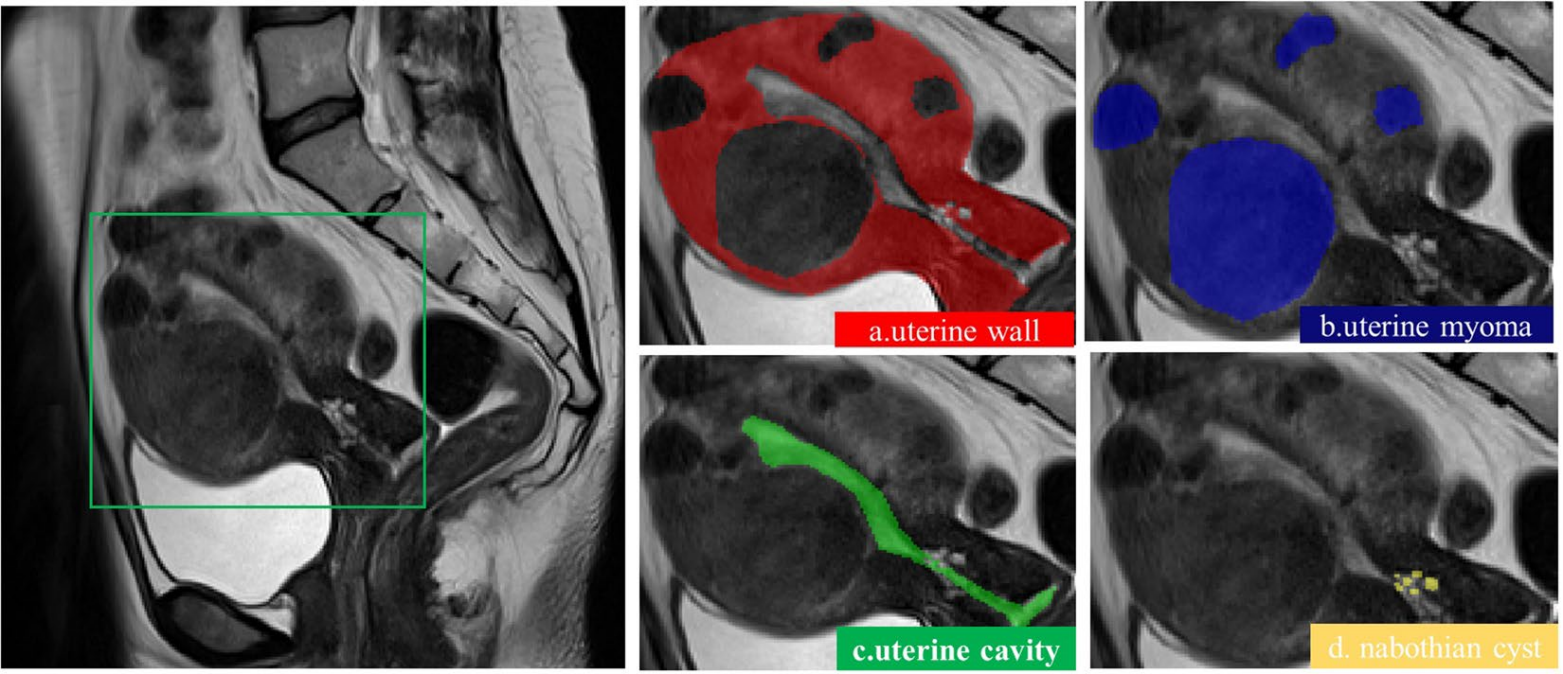
A description of the uterus. Image blocks are shown from left to right.

The UMD includes raw images and annotations with relevant regions, as shown in Fig. 3, which displays the relevant descriptions of the uterus in the T2WI sagittal image.

**Fig. 4:**
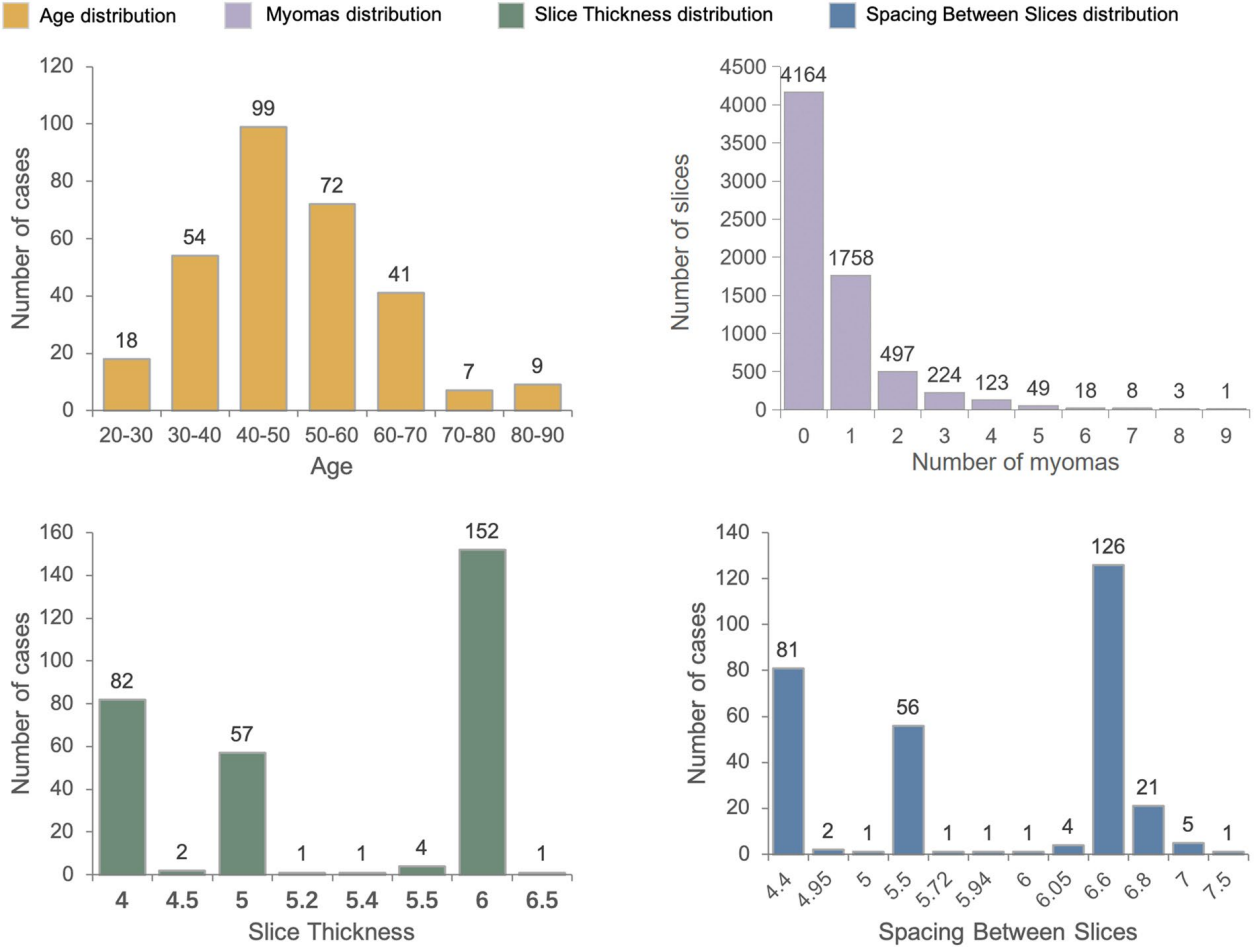
Information statistics at different latitudes in the UMD dataset. **(a)** shows the distribution of age, **(b)** represents the distribution of the number of myomas, **(c)** displays the distribution of the spacing between slices, and Figure (**d**) depicts the distribution of the slice thickness.

Uterine myomas have some impact on human health and life. Image analysis is an effective method for diagnosing this disease. We have provided a uterine myoma MRI dataset consisting of 300 cases, which has been annotated. Detailed information can be found in Table 2, which includes basic information about the UMD dataset. Additionally, we conducted statistical analysis on age, number of myomas, slice thickness, and slice interval for these 300 cases, as shown in Fig. 4.

**Table 2:** The UMD dataset basic information.

Number of cases	Number of slices	Age	Modalities	Category
300	6845	49.73 ± 12.96	T2W	uterine wall, uterine cavity, uterine myoma, nabothian cyst

**Fig. 5:**
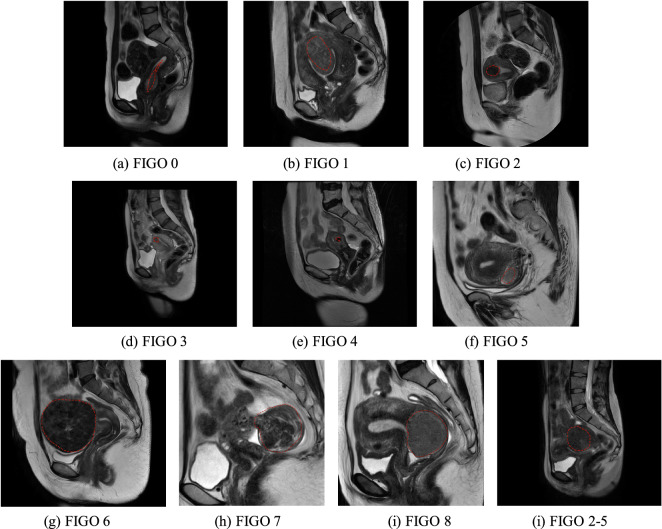
Case examples for different types of uterine myoma. Classifications of myoma according to the FIGO classification system, they are respectively divided into types 0–8, and hybrid myoma(2–5).

The UMD dataset covers 9 different types of uterine myoma (FIGO 0–8) and hybrid myoma according to the FIGO classification system. All patients in the UMD dataset underwent surgical procedures, and all classification results were verified by surgical records and postoperative pathology. As shown in Fig. 5. At the same time, we counted the number of myomas contained in each case data, mainly counting the number of myomas contained in each slice, and the slices with myoma accounted for 36% of all slices. And among these 300 cases of data, one slice contains at most 9 myomas, as shown in Table 3.

**Table 3:** The relationship between the number of myomas and the number of slices.

Number of myomas	Number of slices
0	4164
1	1758
2	497
3	224
4	123
5	49
6	18
7	8
8	3
9	1

## Data Records

**Fig. 6:**
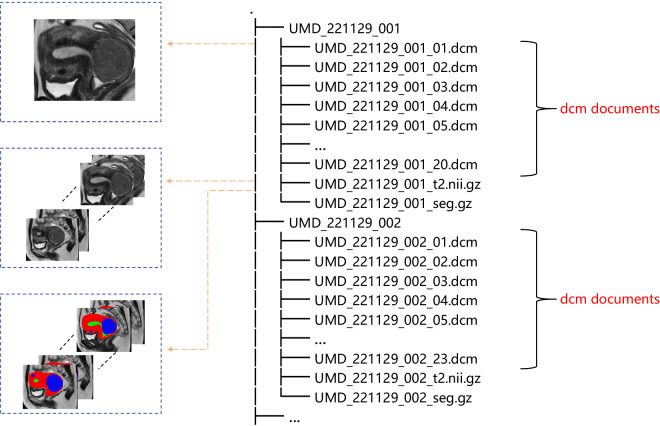
Data types in different formats of UMD dataset. Each case is composed of raw data in DICOM format, raw data in NIFTI format, and labeled data in NIFTI format. (From top to bottom).

The UMD is organized according to the data structure standard of uterine MRI, with a total of 300 cases of data. Each case of data consists of image original data in DICOM format, image original data in NIFTI format, and annotation data in NIFTI format. The directory structure of the UMD is shown in Fig. 6. At the same time, the DICOM header file information carries a lot of information, which can be divided into the following four categories: (a) Patient (b) Study (c) Series (d) Image. We anonymized 300 cases of data provided by UMD, and the data we published only included the anonymized data. The memory capacity of the UMD is 4.12GB. The UMD dataset had been uploaded to Figshare^16^

### Raw data

The original data of female uterine MRI published in this article are T2WI standard sagittal images.

DICOM is saved in the following format: Structural MRI: < UMD_221129_ID > /ID_.dcm.

NIFTI is saved in the following format: Structural MRI: < UMD_221129_ID > /UMD_221129_ID_t2.nii.gz.

### Label data

The UMD dataset is annotated using the ITK-SNAP tool, including four labels, label 1 means uterine wall, label 2 means uterine cavity, label 3 means myoma and label 4 means nabothian cyst. The uterine MRI data designed in this paper is to mark the T2WI sagittal bitmap and save it in the following format: < UMD_221129_ID > /UMD_221129_ID_seg.nii.gz.

The UMD dataset successfully collected all MRI data for each participant. No special circumstances occurred during the data collection process, and no abnormalities were found during further data processing.

## Technical Validation

We evaluated the quality of the dataset from 3 aspects, which are signal-to-noise ratio(SNR), intensity uniformity, and motion artifacts. We will introduce them one by one next.

SNR: Signal-to-Noise Ratio. The image signal-to-noise ratio is a single number obtained by dividing the image signal by the image noise. We used the MRQy^17^ software to calculate the SNR for each MRI data. Based on five different methods, MRQy provides five SNR metrics: PSNR, SNR1, SNR2, SNR3, and SNR4. Referring to the method for calculating SNR based on a single image in the National Electrical Manufacturers Association (NEMA) standard, the calculation method for SNR2:

1 $${\bf{SNR}}2=\frac{S}{{S}_{D}}$$

where *S* refer to the average value of the foreground blocks and *S*_*D*_ refer to the average standard deviation of the background blocks, is the same as the above method, so the SNR2 metric is used to evaluate the data quality.

CJV: Coefficient of Joint Variation. It’s a metric used to assess the level of aliasing and inhomogeneity artifacts in an image. The equation for CJV is given as:

2 $$CJV=\frac{{\sigma }_{F}+{\sigma }_{B}}{\left|{\mu }_{F}-{\mu }_{B}\right|}$$

*σ*_*F*_ and *σ*_*B*_ is the standard deviation of the foreground and background. *μ*_*F*_ and *μ*_*B*_ is the mean of the foreground and background. CJV calculates the sum of the standard deviations of the foreground and background, divided by the absolute difference of their means. A lower CJV indicates better image quality with fewer artifacts.

EFC: Entropy Focus Criterion. It is a metric used to measure the amount of edge information (indicative of focus) in an image.

3 $$EFC=-\sum _{i,j}\frac{F(i,j)}{{F}_{{\rm{m}}{\rm{a}}{\rm{x}}}}{\rm{l}}{\rm{n}}\frac{F(i,j)}{{F}_{{\rm{m}}{\rm{a}}{\rm{x}}}}$$

*F*(*i, j*) is the intensity value at pixel *pixel*(i, j). *F*_max_ is the maximum intensity value in the image.

EFC represents the entropy of the image, which is calculated by summing over all pixels the normalized intensity values multiplied by the natural logarithm of the normalized intensity values. The goal of this measure in medical imaging

**Fig. 7:**
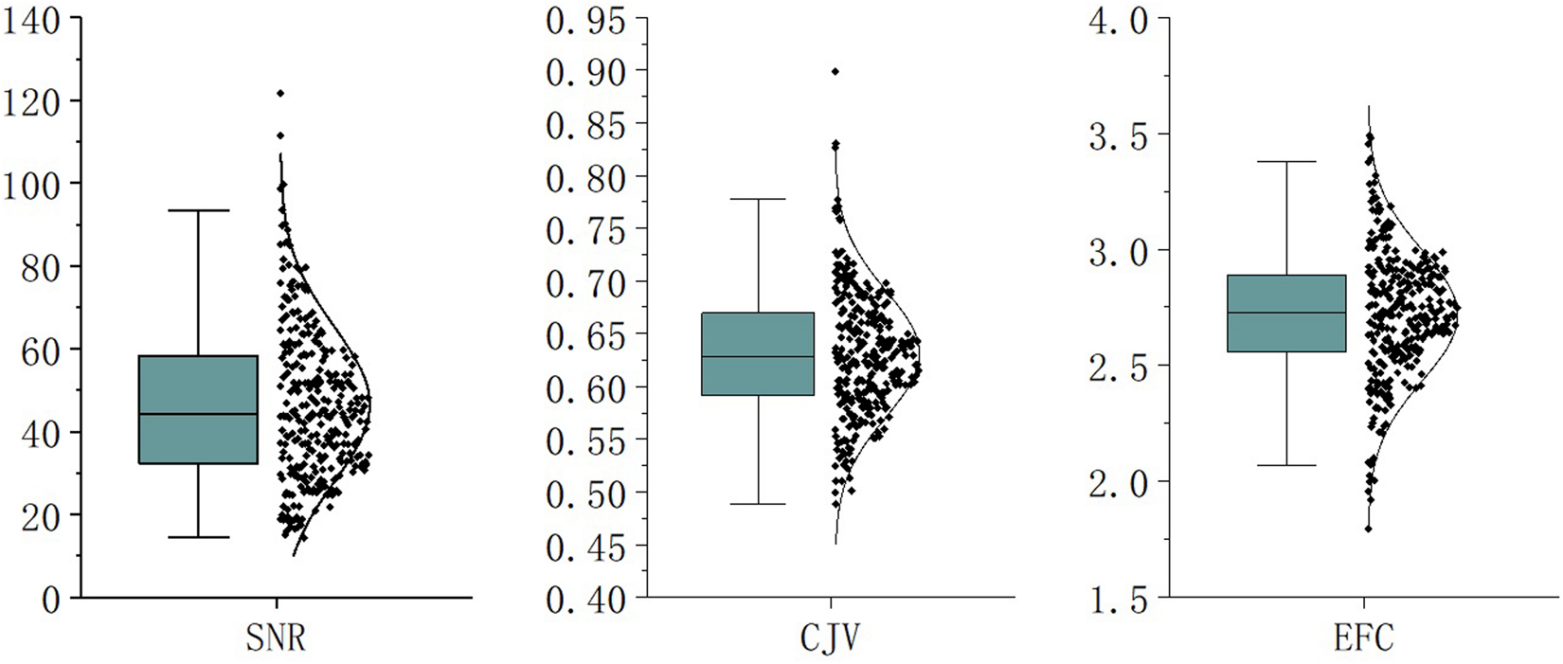
Distribution of SNR and EFC, CJV of MRI data. The SNR actually uses the SNR2 metrics provide by MRQy, and the calculation method is the average value of the foreground block divided by the average value of the standard deviation of the background block. EFC and CJV are also metrics of MRQy output.

The SNR2 values in our dataset range from 14.4 to 121.8, with an average of 46.6. The specific values for SNR, EFC, and CJV data are shown in Fig. 7.

**Fig. 8:**
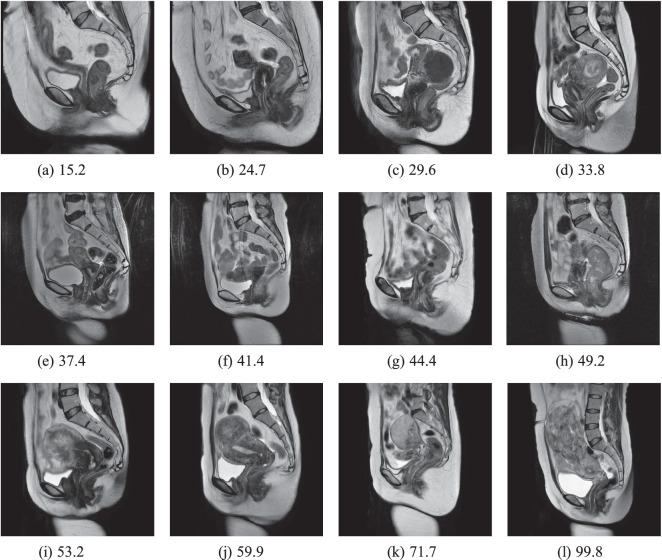
Case examples for different SNR. The SNR keeps getting higher from left to right and from top to bottom(the larger the SNR value, the higher the quality of the MRI).

Based on the calculated SNR2 values, we think that the noise inherent in the data can be ignored. In Fig. 8, we present images of cases from the UMD dataset with different SNR values to reflect the quality of our data.

### Uniformity of strength

Usually, when the MRI intensity is uneven, the quality of the MRI will decrease. Our MRI data collection equipment is regularly inspected to avoid issues of uneven intensity. At the same time, three doctors observed the MRI data to ensure that there was uneven intensity in the data.

### Motion artifact

Motion artifact is caused by voluntary or involuntary movement of the human body, resulting in image blurring. The severity of motion artifact can be evaluated based on visual observation by doctors. Our dataset has been observed by three doctors, who confirmed that the impact of motion artifact is minimal. If not necessary, we prefer to minimize manual processing of the original data, so we did not remove motion artifact from the dataset.

The data we provide has been manually reviewed (subjective evaluation) many times to eliminate inappropriate cases (cases with severe artifacts and cases where foreground and background, object outlines, textures, etc. cannot be distinguished due to other factors), and through professional software (MRQy) for objective assessment. The image quality of the 300 cases we provided is relatively high.

## Usage Notes

As a large-scale dataset, our work obtained MRI images of uterine myomas from 300 patients aged 21–86 years. These data provide a good data foundation and rich data support for artificial intelligence-based clinical diagnosis research of uterine myomas.

Through the study of these data, we can better analyze the distribution and classification of uterine myomas from the perspective of imaging and clinicopathology. The method of artificial intelligence can help clinicians analyze and recognize the imaging performance of uterine myomas. At the same time, it can quantitatively study the classification basis of uterine myomas based on the FIGO classification standard, so as to minimize the experience gap between different doctors.

Although we believe that this dataset is a unique resource for studying the distribution and classification of uterine myomas, we also acknowledge its limitations. The dataset published in this article only focuses on uterine myomas in pathological research and does not target other common gynecological diseases, such as cervical cancer and endometrial cancer. Meanwhile, the data set used in this paper is only studied on the T2W sagittal plane, the image representation under multi-magnetic field information and multi-angle three-dimensional information are not further studied.

## Data Availability

The MRI quality metrics used in this paper are output by the MRQy software, which is available from GitHub (https://github.com/ccipd/MRQy). For DICOM to file to image conversion is the ITK-SNAP tool. Use DICOM Anonymize software to anonymize the sensitive information in the original DICOM header file, and then rename the case.
